# Synaptic inhibitory dynamics drive benzodiazepine response in pediatric status epilepticus

**DOI:** 10.1111/epi.18398

**Published:** 2025-04-15

**Authors:** Tommaso Fedele, Richard J. Burman, Anne Steinberg, Giorgio Selmin, Georgia Ramantani, Richard E. Rosch

**Affiliations:** ^1^ Department of Pediatric Neurology University Children's Hospital Zurich and University of Zurich Zurich Switzerland; ^2^ Institute of Cognitive Neuroscience National Research University Higher School of Economics Moscow Russian Federation; ^3^ Oxford Epilepsy Research Group, Nuffield Department of Clinical Neurosciences University of Oxford Oxford UK; ^4^ Basic and Clinical Neuroscience, Institute of Psychiatry, Psychology and Neuroscience King's College London London UK; ^5^ Wellcome Centre for Imaging Neuroscience University College London London UK

**Keywords:** dynamic causal modeling, EEG, pediatrics, status epilepticus, synaptic inhibition

## Abstract

**Objective:**

Pediatric status epilepticus (SE) is a medical emergency associated with significant morbidity. Benzodiazepines (BZPs) are the current first‐line treatment, but do not work in more than one third of children presenting with SE. Animal studies have shown that SE can cause changes in synaptic inhibition signaling that can ultimately lead to BZPs becoming ineffective. However, the relevance of these mechanisms in pediatric patients with SE remains unknown.

**Methods:**

To test this hypothesis, we combine clinical electroencephalographic (EEG) recordings with dynamic causal modeling (DCM). This approach allows model‐based inference of cortical synaptic coupling parameters based on EEG recorded across distinct oscillatory states.

**Results:**

Our DCM revealed that dynamic changes in inhibitory synaptic coupling explain differences in EEG power spectra associated with BZP treatment responsiveness and guide the transition from ictal to interictal state. Furthermore, in silico simulations demonstrate that there are alternative routes to seizure termination even in cortical circuit models unresponsive to BZPs.

**Significance:**

Together, our findings confirm that alterations in synaptic inhibition underlie BZP response during pediatric SE. More broadly, this work further demonstrates the utility of computational modeling to validate insights from basic science in clinically accessible recordings in neurological disorders characterized by abnormal brain states.

**Key points**

- Changes in synaptic inhibition drive differential responses to treatment with BZPs during pediatric SE.
- DCM provides a link between the preexisting animal work and clinical observations on BZP resistance in SE.
- DCM allows investigation of changes in synaptic parameters underlying routine clinical EEG to evaluate responses to antiseizure medications.

## 1 INTRODUCTION

Pediatric status epilepticus (SE) remains a pertinent clinical challenge. This state of prolonged and unrelenting seizure activity is a medical emergency, as it is associated with significant morbidity in children^1^ as well as being a burden on health care resources.^2^, ^3^ Benzodiazepines (BZPs), available in different subtypes and formulations, are the preferred first‐line treatment due to their ease of administration, fast onset time, and in most cases, effectiveness in terminating SE.^4^, ^5^, ^6^ However, in up to 32% of children presenting with SE, BZPs do not work.^7^ The lack of response to BZP in these children adds complexity to their management, often requiring additional medications and an increased demand on pediatric intensive care unit services, which are often limited, especially in lower–middle‐income countries.^8^, ^9^

Animal studies have demonstrated that underlying the resistance to BZPs are SE‐induced changes in synaptic physiology that alter the main target of these drugs, the γ‐aminobutyric acid type A receptor (GABA_A_R). These chloride (Cl^−^)‐permeable channels play a principal role in mediating inhibitory signaling in the brain and helping restrain aberrant electrical activity such as seizures.^10^, ^11^ During SE‐like activity, there is a cascade of changes that affect the GABA_A_R, which culminate in a loss of intact synaptic inhibition.^7^, ^12^

Despite detailed descriptions of pathophysiological processes in preclinical models of neurological and neurodevelopmental disorders,^13^, ^14^ these insights do not translate to novel treatments in patients.^15^, ^16^ Among possible reasons for this failure, the pathophysiology induced in the preclinical models does not capture the human pathology. This is particularly relevant in etiologically diverse conditions like SE. One essential open question in treatment of refractory SE is therefore whether the inhibitory failure seen in animal models is also a common, shared mechanisms in patients not responding to BZP treatments.

It is here that computational modeling may provide a useful way of translating between the experimental findings and neurophysiological recordings routinely gathered from patients. Modeling the activity of recurrently coupled excitatory and inhibitory neuronal populations is already established for understanding different types of seizure activity, including SE.^17^, ^18^ One such computational framework is dynamic causal modeling (DCM), which has demonstrated utility in studying the hierarchical structure of state transitions in neuronal population activity during epileptic seizures.^19^, ^20^, ^21^, ^22^ An appealing feature of DCM is that it can infer regional disease‐related changes in the cortex, specifically excitatory and inhibitory synaptic transmission between different cortical layers believed to be generating the electroencephalographic (EEG) signal.^23^, ^24^ In this way, DCM offers an opportunity to study how changes in synaptic dynamics contribute to disease pathophysiology, which is highly relevant in the context of BZP‐resistant SE.

Here, we combine scalp EEG recordings with DCM to explore the mechanisms underlying BZP resistance in pediatric SE. This is a unique cohort for whom drug administration during SE was captured by continuous EEG monitoring as part of routine clinical care. Through our analysis of this dataset, we demonstrate how changes in synaptic inhibition differentiate children who are unresponsive to BZPs. We also reveal that patients who are resistant to BZPs appear to have both preexisting and acquired electrographic features that appear to affect their sensitivity to BZPs.

## 2 MATERIALS AND METHODS

### 2.1 Patient selection

This study used retrospective anonymized patient data and was approved by the local ethics committee (Kantonale Ethikkommission Zürich, KEK‐ZH PB‐2020‐02580). A consent waiver was granted. We retrospectively identified pediatric patients with SE (<18 years of age) who underwent scalp EEG recordings between July 2008 and February 2020 at the University Children's Hospital Zurich. Our inclusion criteria were electroclinical diagnosis of SE, and administration of an antiseizure medication (ASM) during the EEG recording. Neonatal patients (<1 month of age) were excluded. The sample includes only patients who have been connected to EEG prior to the BZP administration. This includes patients who did not completely respond to first‐line BZP given for convulsive seizures, or who may have been connected to EEG for possible nonconvulsive seizures, where EEG was necessary to confirm diagnosis. Appropriate dosing and administration of BZPs were reviewed and confirmed to align with our institutional guidelines.

### 2.2 EEG acquisition and analysis

Clinical EEG recordings (21 electrodes, international 10–20 electrode layout, 256‐Hz sampling frequency, 1–70‐Hz band pass) were visually reviewed and annotated by two EEG reviewers with relevant training in epileptology and clinical neurophysiology (G.S. and G.R.), and discrepancies were resolved by consensus. Annotations included (1) characterization of electroclinical features including (i) onset and offset timestamps for individual seizures or ongoing ictal activity, (ii) classification as convulsive/nonconvulsive (subclinical), (iii) classification as continuous or intermittent, and (iv) classification as focal or generalized^25^; and (2) characterization of drug treatments including (i) drug administration timestamps and (ii) classification as first‐line (i.e., BZPs; e.g., diazepam, lorazepam, midazolam), second‐line (i.e., different ASM; e.g., levetiracetam, phenobarbital, valproate), or third‐line (i.e., anesthetics; e.g., propofol; for a synthetic overview, see Table S1; for more details, see Table S6).

All EEGs clinically reported as showing SE were screened for notes that indicate administration of ASM. Patients with clinically unequivocal SE typically received first‐line treatment before EEG recording was initiated. Therefore, the sample presented here comprises patients who did not respond to first‐line treatment and presented clinical semiology necessitating EEG confirmation. Of these, 17 records were identified that captured administration of a short‐acting BZP with the aim to acutely stop ongoing ictal EEG patterns. All these were included in the analyses.

In a second step, we classified each individual BZP medication administration captured on EEG based on electrographic changes following BZP administration into "response" and "no response." This classification was made based on EEG features alone, without reference to clinical parameters, and confirmed independently between two EEG reviewers. Where there was disagreement in classification, consensus was reached through discussion. This classification was made before any quantitative analysis.

With this approach, we select for patients with EEG changes in the responder group and those without in the nonresponder group; however, we do not specifically bias the analysis toward differences between group prior to drug administration.

To evaluate the effect of drugs on the EEG, we identified 5‐min artifact‐free EEG intervals preceding and following drug administration: *premedication intervals*, terminating 1 min before drug administration, and *postmedication intervals*, starting 6 min after drug administration in nonresponders and 1 min after the termination of the continuous SE or the last seizure in intermittent SE in responders. Six minutes was an empirical choice guided by an average response time to BZPs of 340 ± 40 s (mean ± SE) in our cohort (for more details, see Table S5). We did not observe changes in the EEG spectral power for several minutes after SE termination in responders (see Figure S4).

The recording length available prior to BZP administration varied based on clinical circumstances, including in‐hospital onset of SE and EEG availability. A 5‐min duration was pragmatically chosen to capture stable pre‐ and postadministration spectral features while maximizing patient inclusion in the study.

### 2.3 EEG statistical analysis

To quantify group‐level differences in the pre‐ and postmedication EEG spectra of responders and nonresponders to BZP, we used a repeated‐measures analysis of variance using the factors treatment time (pre‐ vs. postmedication) and treatment efficacy (responders vs. nonresponders). Post hoc analysis was performed with Student *t*‐test (Bonferroni correction, *n* = 6 [four groups], significance at uncorrected *p* = .0083).

### 2.4 EEG dimensionality reduction

We reduced the dimensionality of the data through an independent and identically distributed source localization algorithm (SPM12) with the aim to capture each patient's characteristic ictal rhythms in a single time series, analogous to previously published approaches.^24^, ^26^ For this, representative 30‐s artifact‐free ictal segments were visually identified from the EEG window prior to drug administration to generate an anatomical activation map that captures key cortical sources contributing significantly to ictal activity. Based on the cortical location with the highest amplitude activation, we reconstructed the time course of the corresponding virtual local field potential (vLFP) for the entire pre‐ and postmedication segments. The anatomical location of the vLFP for each patient is provided in Table S1, based on Automated Anatomical Labeling.^27^ This vLFP approach reconstructs signals at single points on the cortical mesh of a three‐layer standardized head model using an empirical Bayes beamformer.^28^ The vLFP signal and its power spectra closely resembled the activity of the EEG channels best capturing the ictal patterns (see Figure S3).

### 2.5 Dynamic causal modeling

To infer synaptic parameters governing neuronal populations' ongoing oscillatory activity, we used a DCM approach—specifically, DCM for cross‐spectral densities in electrophysiological data.^29^, ^30^ Briefly, DCM is a Bayesian model inversion framework in which parameters of dynamic models are fitted to time series data using a variational Laplace approximation. Typically,^26^, ^31^, ^32^ modeling of oscillatory electrophysiological activity is performed by fitting synaptic coupling parameters of neural mass population models to cross‐spectral summaries of the presumed stationary time series. This approach builds on an expansive body of work, framing the dynamics of EEG recordings as summary measures of neuronal population dynamics,^33^, ^34^, ^35^, ^36^ that can be described through a small set of parameters describing the strength and temporal dynamics of excitatory and inhibitory neuronal coupling.^37^ Neural mass models summarize the activity of functionally distinct groups of neurons as point processes and their average interaction as synaptic parameters through a mean field approximation, thus allowing the modeling of macroscopic electrophysiological data with tractable parameter numbers.

Here, we fitted single source models to dimensionality‐reduced single channel representations of EEG activity pre‐ and postmedication for each patient individually.^38^ Here, we assume that the single EEG trace representative of regional activity is generated by the combined activity of four recurrently coupled neural masses—the canonical microcircuit (CMC)^39^, ^40^—describing layered cortical populations as recurrently coupled sets of excitatory and inhibitory cortical sources (Figure 2C).

Although the CMC is specifically designed to capture layered cortical architecture, it effectively models pairs of coupled oscillators, accommodating a range of resonant frequencies. This versatility enables the CMC to model a broad spectrum of both cortical^41^ and noncortical^42^ activity.

These model inversions yield two types of output. The first is optimized estimates for the synaptic parameter combinations that yield the kinds of dynamics observed in the EEG data; formally, these are Bayesian posterior densities that encode the estimate, and uncertainty around the specific parameter value. These parameter estimates can then be used to simulate EEG data and explore the effects of slight perturbation of the identified parameter set on the EEG spectrum.^32^ Second, the inversion also provides a Bayesian estimate of the model evidence, approximated through the model free energy. Because this represents the evidence for a given model parameterization, given the data, the free energy can be used to directly compare the evidence a given dataset provides for competing model parameterizations, providing statistical estimates of which model is a better explanation for the observed dataset, while balancing model accuracy and model complexity.^43^

### 2.6 First‐level DCM

We then fitted spectral patterns in the vLFP before and after drug administration using an adapted convolutional CMC model within the DCM framework. Here, parameters are fitted for a model of a single cortical column consisting of four neuronal populations (superficial pyramidal cells, spiny stellate interneurons, inhibitory interneurons, deep pyramidal cells), which are coupled through recurrent excitatory and inhibitory connections.^20^ The dynamics of our adapted whole microcircuit is described by 14 key variable parameters that determine the time constants of their convolutive kernel (four parameters), and the intrinsic excitatory (three parameters), inhibitory (three parameters), and self‐modulating (four parameters) coupling (Tables S2 and S3).^20^ For each patient, two separate models were fitted: one parameterized to reproduce the premedication spectrum and one for the postmedication spectrum. These models were fitted to a broadband frequency spectrum (1–45 Hz) and adapted to suppress changes in nonneuronal scaling parameters within repeat inversions of models for the same patient.

### 2.7 Group‐level statistical inference over dynamic causal models

As a next step, we wanted to identify which synaptic parameters are consistently changed by the effect of BZP administration and how the difference between responders and nonresponders arises. We addressed this using a parametric empirical Bayesian approach,^44^ which allows inferring effects across individual DCMs.

Here, we used this approach specifically to identify first whether responders differ from nonresponders in their particular synaptic coupling during SE, their response to BZP, or both. Second, we wanted to identify which subset of synaptic parameters best describes both the BZP‐associated changes and the responder versus nonresponder differences. This was tested by designing different hierarchical models where between‐DCMs are captured in different design matrices and comparing them using free energy estimates of the model evidence at this second level.

The hierarchical modeling approach allows integrating a user‐specified design matrix to link (1) "first‐level" dynamic causal models that model separate neurophysiological datasets (e.g., data from individual patients) with (2) "second‐level" hierarchical linear models that model systematic variation of the first‐level model parameters (e.g., synaptic parameter differences between patients groups). These hierarchical models are inverted in a Bayesian framework that allows for propagating uncertainty over model parameters from the first to the second level, as well as optimization of first‐level parameter based on iterative second‐level parameter estimates.

We used the two first‐level DCMs per patient that capture the pre‐ and postmedication window, and structured a second‐level design matrix to estimate the across‐DCM effects of whether the patient turned out to be a responder to BZPs (the "main effect of responsiveness"), the treatment with BZPs (the "main effect of BZPs"), and an interaction term capturing conditional effects of BZPs specific to patients who ultimately respond (the "interaction term"). Treatment and interaction terms were included also for other ASMs and considered as random effects. Details on the structure of the design matrix adopted for the hierarchical model inversion are provided in Figure S1.

At the same time, we wanted to identify subsets of synaptic parameters that would provide the more parsimonious explanation for the observed differences both within‐subject (pre‐ vs. postmedication administration) and between‐subjects (responders vs. nonresponders). For this, we created a model space where only subsets of the synaptic model parameters were allowed to vary during the model inversion, divided parameters into functional classes (excitatory coupling, inhibitory coupling, modulatory recurrent self‐connections, synaptic time constants), and comprehensively tested all combinations (i.e., 15 individual models). We then performed a fixed effects Bayesian model comparison across these second‐level models, using the free energy approximation of the model evidence.^36^

Effectively, there are four conditions modeled through our analysis, corresponding to a two‐by‐two factorial design: recordings pre‐ and posttreatment in the patients who respond and do not respond to BZPs. These are modeled through the two main effects (treatment, group) and an interaction term (treatment × group). The parametric empirical Bayesian inference allows identification of synaptic parameter‐level differences associated with these group‐level effects. Therefore identifying, for example, the BZP effect relies on shared parameter differences in responders and nonresponders that are not already explained away by the interaction term in the model.

### 2.8 Simulations of effects of individual synaptic parameters

The DCM framework allows extraction of optimal values of synaptic parameters fitting a given power spectrum. Conversely, we can simulate the effect of altering parameter values on the power spectrum. First, we identified initial conditions for the synaptic parameters by fitting a first‐level DCM to the average spectra of premedication responders and nonresponders separately. Second, we systematically altered the value of synaptic parameters of choice, generating corresponding altered power spectra. We devised simulations aimed to reproduce in silico the group‐level effects extrapolated by the second‐level DCM. Additionally, we performed a fully comprehensive parameter screen across the 14 synaptic parameters (Figure S2). In this way, we identified parameters that allow for nonresponders to "escape" the ictal power spectral patterns.

## 3 RESULTS

### 3.1 Differences in spectral features characterize variable responses to BZPs in pediatric SE

**FIGURE 1:**
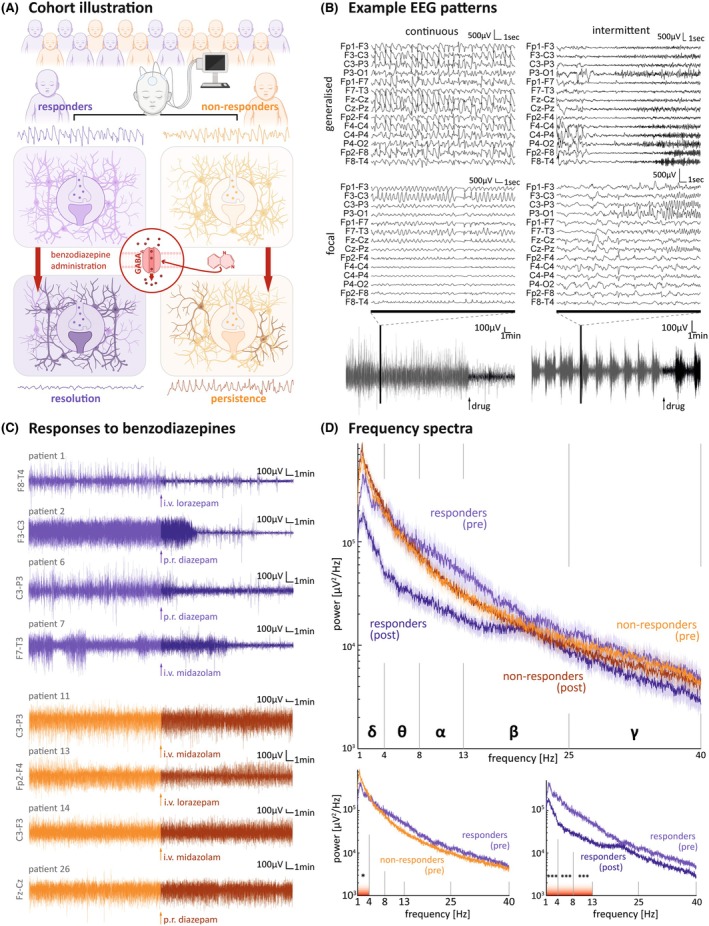
Differences in spectral features characterize variable responses to benzodiazepines (BZPs) in pediatric status epilepticus. (A) Schema of the study design in which EEG recordings were acquired from a cohort of patients (*n* = 26) who received BZPs during an episode of status epilepticus (SE; *n* = 17). BZPs are γ‐aminobutyric acid type A (GABA_A_) receptor allostatic modulators and expected to enhance synaptic inhibition. Patient whose SE was terminated by BZPs were labeled "responders" (purple, *n* = 8), whereas those whose SE persisted after BZP treatment were labeled "nonresponders" (orange, *n* = 9). (B) Raw sample electroencephalographic (EEG) traces of different electrographic presentations of pediatric SE with different spatial (i.e., generalized vs. focal) and temporal (i.e., continuous vs. intermittent) features that were included in the analysis. Bottom panel illustrates zoomed‐out view of single electrode recording and demonstrates period before (gray) and after (black) BZP administration (arrows). (C) Raw sample EEG traces demonstrating both responsive patients (top panel, purple) and nonresponsive patients (bottom panel, orange) before (lighter shade) and after (darker shade) different administrations and formulations of BZPs. (D) Spectral analysis of EEG traces (mean ± SEM) reveals difference in power spectra between responders and nonresponders before (pre) and after (post) BZP administration in the theta band (dark line illustrates mean, shade illustrates the SEM, *F* = 1.85, *p* = .02, two‐way repeated measures analysis of variance). Across all patients, before BZP administration (bottom left panel) there was a significant difference in delta band power between responders and nonresponders (responders: 2.74e5 ± 3.1e4 μV^2^/Hz vs. nonresponders: 4.27e5 ± 4.5e4 μV^2^/Hz, *p*_*corr*_ < .001, Student *t*‐test). In responders, BZP administration significantly changed the spectra in the delta (responders pre: 2.74e5 ± 3.1e4 μV^2^/Hz vs. responders post: 1.12e5 ± 1.27e4 μV^2^/Hz, *p*_*corr*_ < .001, Student *t*‐test), theta (responders pre: 1.37e5 ± 1.53e4 μV^2^/Hz vs. responders post: 3.72e4 ± 4.16e3 μV^2^/Hz, *p*_*corr*_ < .001, Student *t*‐test), and alpha (responders pre: 7.06e4 ± 7.9e3 μV^2^/Hz vs. responders post: 2.3e4 ± 2.57e3 μV^2^/Hz, *p*_*corr*_ < .001, Student *t*‐test) frequency bands. *p < .05, ***p < .001. i.v., intravenous; p.r., per rectal; α, alpha band (8–13 Hz); β, beta band (14–30 Hz); γ, gamma band (>30 Hz); δ, delta band (1–4 Hz); θ, theta band (4–8 Hz).

We analyzed the EEG recordings of pediatric patients with SE treated with BZPs and other ASMs (Figure 1A,B, Table S1). Among patients treated with BZPs, those who showed a clear resolution of the SE on visual EEG interpretation were labeled responders, whereas those in whom SE persisted were labeled nonresponders (Figure 1C). The modulation of the EEG was reflected in the power spectra of extracted vLFPs used for DCM analysis, which revealed a significant effect of BZP treatment across responders versus nonresponders to BZP (Figure 1D). Post hoc analysis revealed significant differences between responders and nonresponders in premedication delta power. In responders, the pre‐ to postmedication delta–alpha range spectral power differed significantly, whereas in nonresponders no difference was detected. There was no difference in the rate of continuous versus intermittent electrographic patterns between responders and nonresponders (responders: seven continuous, one intermittent; nonresponders: seven continuous, two intermittent; chi‐squared test, *p* > .05).

### 3.2 DCM enables estimation of changes in synaptic parameters

**FIGURE 2:**
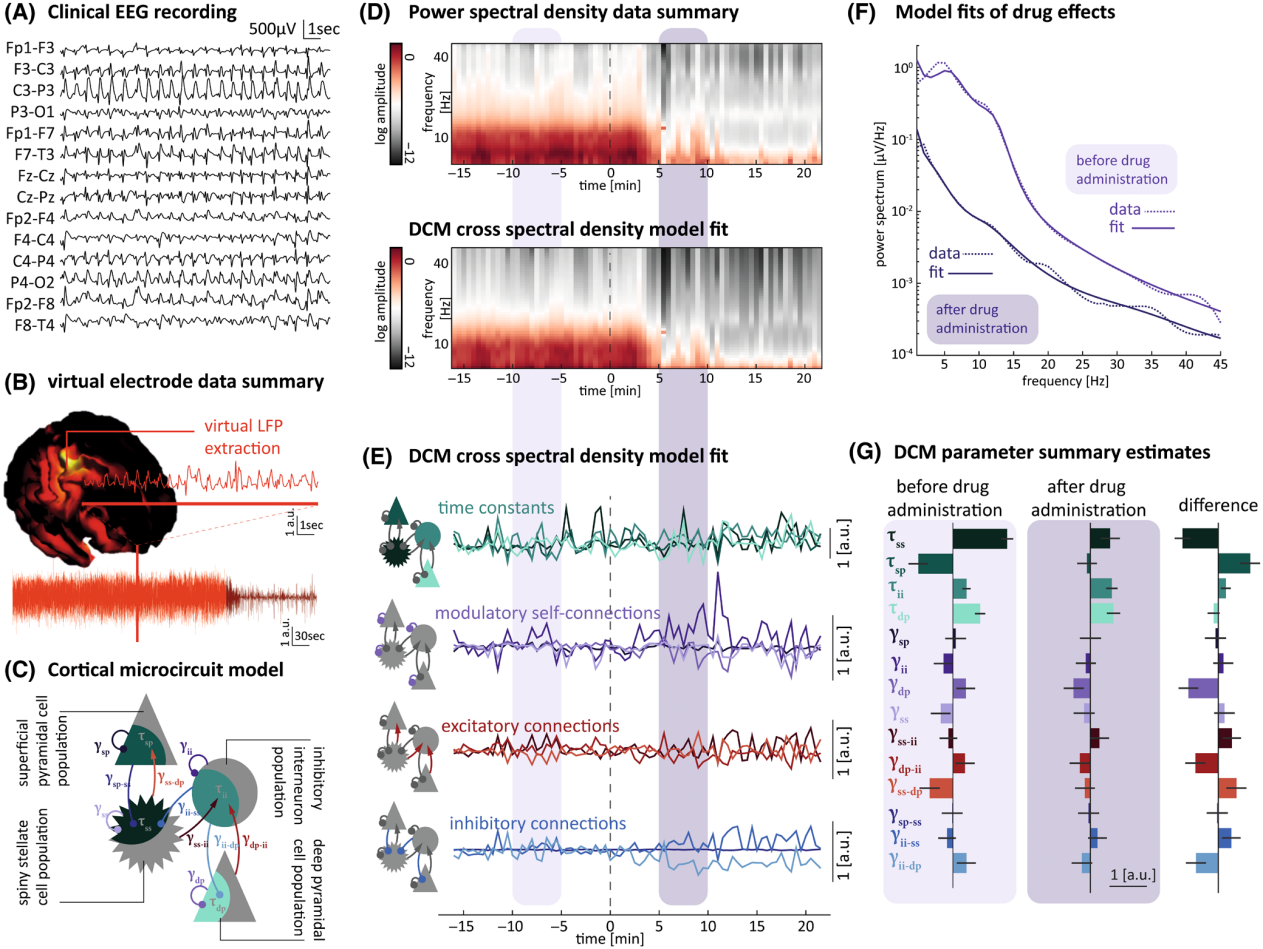
Dynamic causal modeling enables estimation of changes in synaptic parameters from clinical recordings of pediatric status epilepticus. (A) Example raw electroencephalographic (EEG) trace from a single patient (responder), which is then used to extract a virtual local field potential (LFP; B) that can then be used to study the effect of benzodiazepine (BZP) administration. Red line highlights zoomed‐in view of trace. (C) Schematic of canonical microcircuit model of a cortical column, consisting of four coupled neural masses, representing layered populations capturing key aspects of intracortical connectivity and population dynamics. (D) Time‐resolved power spectral density data (30 s) from the virtual LFP before (top panel) and after being fitted by dynamic causal modeling (DCM; bottom panel). Five‐minute epochs before (light purple) and after (dark purple) BZP administration (dashed line) were used in parameter analysis. (E) Time series of changes in cortical microcircuit physiological parameters including time constants (green), modulatory self‐connections (purple), excitatory connections (red), and inhibitory connections (blue). (F) Spectral analysis of virtual LFP trace from 5‐min epochs before (light purple) and after (dark purple) BZP administration highlighted in panel D. Empirically recorded (dashed lines) and DCM‐fitted data (solid lines) are superimposed for pre‐ and postmedication windows. (G) Summary of changes in cortical microcircuit physiological parameters before and after BZP administration for a single patient (each parameter value expressed as expectation ± variance). τ, time constant; γ, connection strength; dp, deep pyramidal cells; ii, inhibitory interneurons; sp, superficial pyramidal cells; ss, spiny stellate cells.

Next, we fitted the vLFP power spectra with a neural mass model through DCM. From the clinical EEG (Figure 2A), we extracted the vLFP best representing SE (Figure 2B). The core component of the DCM is the CMC (Figure 2C).^39^ The CMC includes superficial (supragranular) pyramidal neurons, spiny stellate excitatory interneurons, inhibitory GABAergic interneurons, and deep (infragranular) pyramidal neurons. Connections indicate synaptic connectivity parameters that can be excitatory (red), inhibitory (blue), or self‐modulatory (purple). Neuronal activity is further characterized by time constants (green) governing the response of populations to input. These generative models are used to estimate the contribution of different excitatory and inhibitory synaptic parameters to observed EEG patterns (see Table S2). DCM can be fitted to the vLFP power spectra for different time windows and allow for synaptic parameters to be extracted (Figure 2D). These parameters are functionally grouped here as time constants as well as excitatory and inhibitory coupling (Figure 2E). For subsequent group‐level analysis, we included a single pre‐ and postmedication time window for each patient. This yielded two first‐level DCMs for each patient, fitting the pre‐ and postmedication power spectra (Figure 2F). Model fits for single spectra were overall robust across patients, showing a correlation of .80 ± .15 between observed and predicted spectral densities. Model inversion further provided estimates of synaptic parameters (Figure 2G).

### 3.3 Synaptic inhibition during SE differs between BZP responders and nonresponders

**FIGURE 3:**
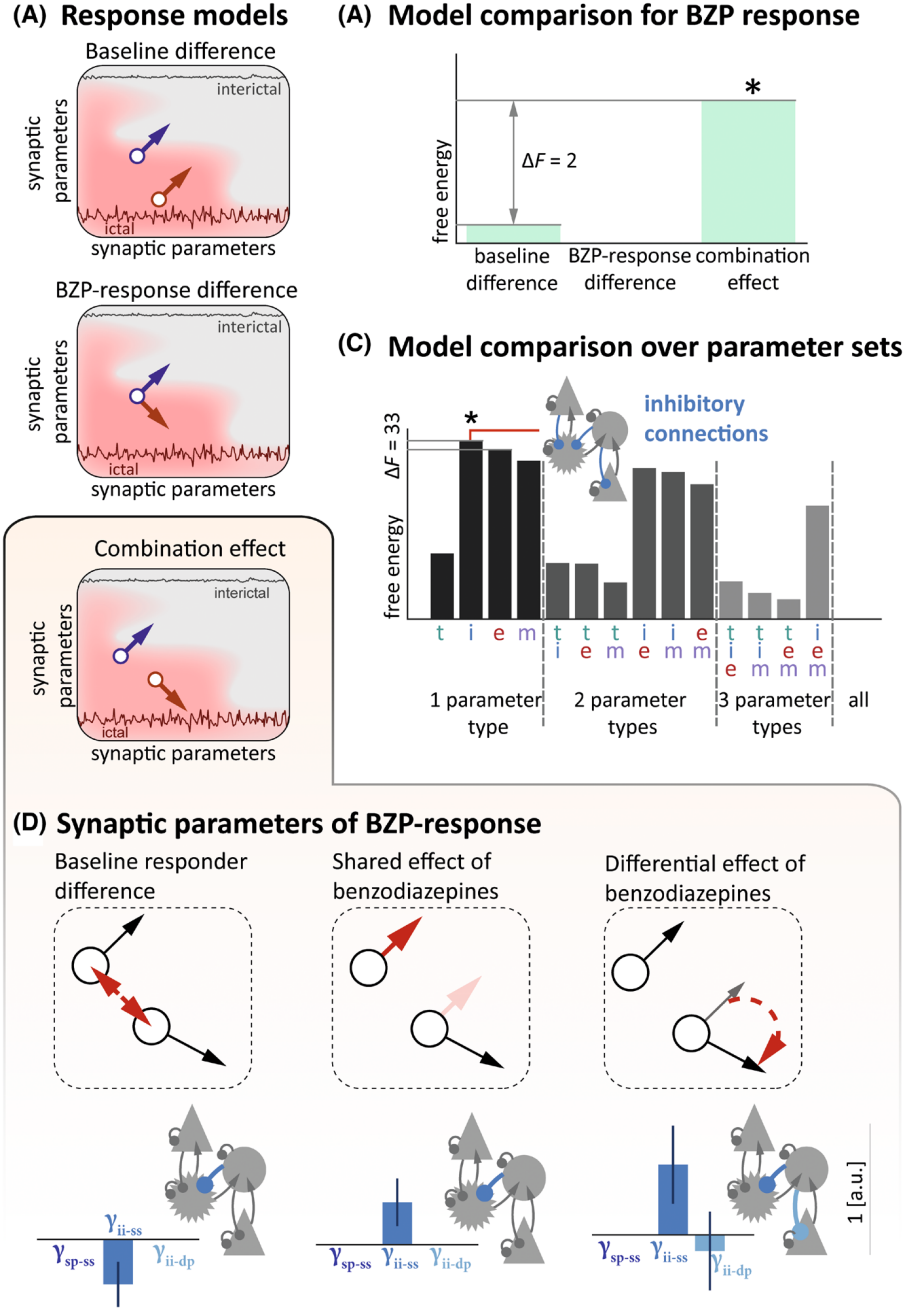
Benzodiazepine (BZP) response is determined by both inherent and acquired differences in inhibitory synaptic coupling. (A) Models space for the difference between responders and nonresponders, where their synaptic parameters (top panel) differ during SE, but get similarly altered by BZPs; (middle panel) are similar during status epilepticus (SE) but are distinctly altered by BZPs; and (bottom panel) both differ in SE and are distinctly altered by BZPs. Across these models, the premedication state is indicated by a circle, the BZP effect is indicated by an arrow; orange signifies nonresponders, and purple signifies responders. (B) Relative free energy calculated for each of the three hierarchical models revealing that the third, combined model best explains the variability in the electroencephalographic signals in our patient cohort. (C) Relative free energy for a set of models in which this combined effect of BZP treatment across responders and nonresponders is modeled only with subsets of microcircuit parameters, indicated as t (time constants), m (self‐modulatory coupling), e (excitatory coupling), and i (inhibitory coupling); multiple letters indicate combinations of these parameter sets. The model based on changes only in inhibitory coupling provided the best explanation for BZP treatment effects and the difference between responders and nonresponders. (D) Synaptic parameters inferred in the winning model; inhibitory parameter values are shown for each of the constituent effects making up the winning model from panel A, where nonresponders differ from responders during SE and are differently altered by BZPs. This is described in three main constituent effects indicated in the red arrows: (1) a difference during established SE, (2) a common response to BZP, and (3) a selective response to SE in responders. These constituent effects are shown as red arrows; the full difference being inferred is shown as black circles and arrows. *Winning model (highest free energy).τ, time constant; γ, connection strength; dp, deep pyramidal cells; ii, inhibitory interneurons; sp, superficial pyramidal cells; ss, spiny stellate cells.

We inferred which synaptic coupling parameters underlie group‐level differences in spectral power across pre‐ and postmedication time windows (within subjects) as well as across responders and nonresponders (across subjects). For this, we employed hierarchical DCMs, considering the effect of responsiveness, BZP treatment, and their interaction as a second‐level design matrix (Figure S2). We formulated three main hypotheses regarding the between‐subject effect of BZP responsiveness (Figure 3A). Hypothesis 1 is that synaptic parameters in responders and nonresponders differ during SE but are similarly altered by BZPs; hypothesis 2 is that parameters are similar during SE but are distinctly altered by BZPs; and hypothesis 3 is that parameters differ already during SE and are also distinctly altered by BZPs.

To test these hypotheses, we inverted second‐level models with subsets of the possible effects included in the full design matrix, where hypothesis 1 is modeled with a design matrix comprising the "main effect of responsiveness" and the "main effect of BZPs"; hypothesis 2 is modeled with a design matrix comprising the "main effect of BZPs" and the "interaction term"; and hypothesis 3 is modeled with a full complement of the "main effect of responsiveness," the "main effect of BZPs," and the "interaction term." When comparing the free energy for these models, hypothesis 3 was the winning model. In a second step, we wanted to further test whether a subset of synaptic parameters is sufficient to capture the within‐subject and between‐subjects effects. Models only allowing variation in inhibitory parameters showed the highest evidence (Figure 3B). Among these models, the one addressing hypothesis 3 revealed that, at the microcircuit level, responders exhibit a lower level of GABA‐mediated inhibition on spiny stellate cells during established SE (γ_ii‐ss_, Figure 3D, left panel); BZPs result in increased GABA‐mediated inhibition on spiny stellate cells (γ_ii‐ss_, Figure 3D, middle panel); in addition, there is a further BZP‐mediated increase in GABA‐mediated inhibition on spiny stellate cells in the responders (γ_ii‐ss_), coupled with a slight decrease in deep pyramidal cell inhibition (γ_ii‐dp_, Figure 3D, right panel).

These findings suggest that the variability in the spectral modulation observed between BZP responders and nonresponders in this cohort can be primarily attributed to the neural inhibitory drive (Figure 3C,D, Table S4).

### 3.4 Probing of synaptic parameters reveals alternative mechanisms to terminate SE

**FIGURE 4:**
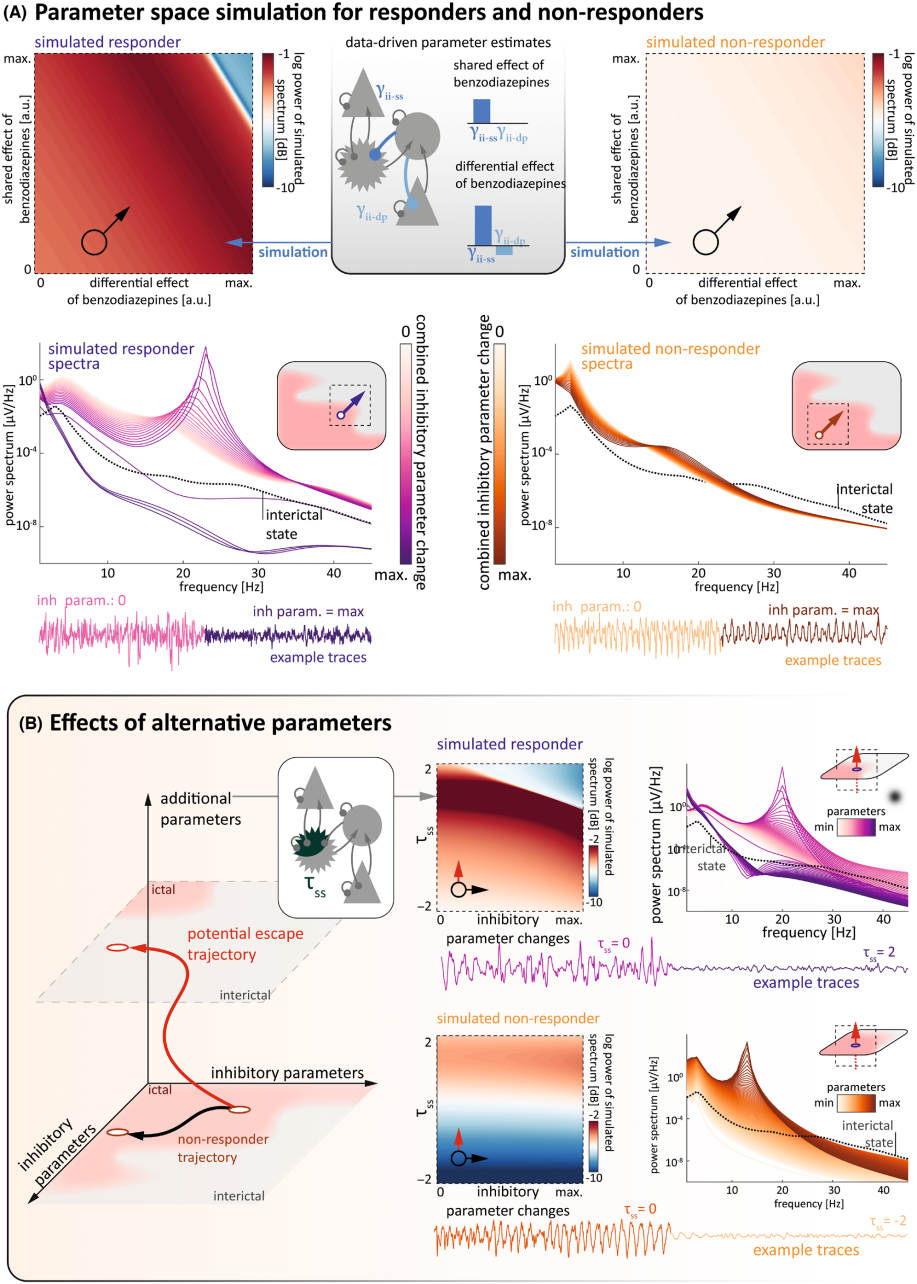
In silico probing of synaptic parameters reveals alternative mechanisms to terminate status epilepticus. (A) Power spectra (at 25 Hz) generated from a hierarchical parameter empirical Bayes model (top panel, middle) demonstrating the relationship between the shared and differential response to benzodiazepines (BZPs) and their combined effect on directing a transition from status epilepticus (SE; a continuous ictal state) to termination (an interictal state). These simulations used parameters extracted from dynamic causal modeling (DCM) fitted to data from the responder (top panel, left) and nonresponder (top panel, right) patient groups to reproduce changes in inhibitory coupling retrieved from DCM. A starting value was assigned to DCM parameters in responders and nonresponders (to BZP). Bottom panel shows the power spectra along the diagonal trajectory (bottom left to top right, across inhibitory parameters [inh param.] and frequencies) from each of the corresponding power spectral plots in the top panel. The trace under the plot reflects the simulated electroencephalographic (EEG) signal generated from the spectral properties observed. In the responder group, there is a discontinuity in the spectral power, which corresponds with the transition from an ictal state (i.e., in SE) to an interictal state (i.e. SE termination) as shown in the insert. By contrast, in nonresponders this transition is not present and reflects that these patients are further away from the termination point (insert). Different color coding between the simulated responder (left) and nonresponder (right) reflect spectra generated from different regions of the parameter space. (B) Schematic illustrating how this in silico approach to probing synaptic parameters can be used to reveal addition parameters that may contribute to terminating SE, such as the time constant of spiny stellate cells (τ_ss_; left panel). With the addition of τ_ss_, it is possible to reproduce power spectra for the responders and nonresponders by relating changes in τ_ss_ with changes in inhibitory parameters (middle and right panels). This demonstrates how manipulating τ_ss_ can push SE in both the responders and nonresponders toward termination, as shown in simulated EEG traces. τ, time constant; γ, connection strength; dp, deep pyramidal cells; ii, inhibitory interneurons; sp, superficial pyramidal cells; ss, spiny stellate cells.

The estimated hierarchical model identifies relevant contributions of microscale dynamics influencing the transition from SE to seizure termination following BZP administration. To validate our hierarchical model in delineating the conditions for state transition, we simulated power spectra mimicking the pathophysiological state prior to ASM administration in responders and nonresponders and then navigated the parameter space along the shared and differential effects of BZP identified by the model. In silico state transition from the ictal to interictal state was observed only in responders, where no qualitative change was observed in nonresponders (Figure 4A). Based on a sensitivity analysis on the single synaptic parameter level (Figure S2), we identified alternative scenarios, where the shift in additional parameters suggests alternative possible pathways for state transition also in nonresponders (Figure 4B), which are qualitatively distinct from those in responders.

## 4 DISCUSSION

Our study provides evidence that pathological alterations of synaptic inhibition underlie BZP failure in pediatric SE. We demonstrated that spectral changes observed in clinical EEG following BZP administration are associated with the dynamic modulation of GABA‐mediated inhibitory coupling in generative models of cortical dynamics. Furthermore, EEG spectral power showed two main effects in our cohort. First, responders to BZP treatment exhibited reduced low‐frequency spectral power prior to treatment, which may indicate the electrographic signature of less prolonged SE. Second, responsiveness to BZPs was associated with a wideband decrease in neural synchronization,^45^ a well‐documented effect following the potentiation of the GABAergic transmission.^46^, ^47^

Our computational framework successfully captured the differences in excitatory–inhibitory coupling between responders and nonresponders to BZP and detected a shift in inhibitory GABA‐related drive following BZP administration. This is in line with experimental data suggesting that alterations in GABA‐mediated inhibition underlie changes in BZP responsiveness.^7^ First, our results confirm the involvement of the inhibitory system in the pathophysiology of BZP failure. This result is in line with those from the animal literature,^12^, ^48^, ^49^ suggesting that similar common mechanisms of inhibitory failure contribute to treatment resistance in etiologically diverse cases of SE in patients. Additionally, understanding the nature of the inhibitory failure is essential. Preclinical models have identified both preexisting changes in inhibition and abnormal responses to BZPs as mechanisms toward nonresponding in episodes of SE.^7^ Both mechanisms have been identified in this study, suggesting that novel treatments need to target noninhibitory mechanisms and in the future may be stratified early due to initial spectral EEG changes in response to first‐line BZP treatment.

Our findings further demonstrate that this type of synaptic inhibition plays a crucial role in modulating the observed EEG dynamics in pediatric patients with SE and further build on the use of generative models to characterize synaptic mechanisms underlying EEG responses.^50^

The transition between dynamic states emerges from the slow fluctuation of nonlinear evolving phenomena. Dynamic models have described the transition between seizures and physiological state in terms of critical transition and bifurcation analysis, inspired by and validated on experimental evidence.^18^, ^51^, ^52^ We successfully validated in silico the role of the GABAergic drive in the transition to seizure termination. Notably, the enhancement of inhibitory coupling had a specific effect in a region of parameter space that aligned with the premedication EEG spectrum observed in responders, reproducing the spectral modulations typically observed after BZP administration.^53^ These findings underline the intricate relationship between microscale dynamics and macroscale brain activity, illustrating how subtle alterations in synaptic interactions can have profound effects on overall brain function in these complex dynamic systems. Importantly, generative models allow further interrogation of the dynamic landscape through simulations. Here, the computational framework showed alternative potential pathways in parameter space leading to SE termination, which were not observed in our dataset. In this context, DCM has the potential to prioritize potential alternative treatment strategies for empirical investigation and in the future to provide noninvasive monitoring for alternative therapeutic strategies. There is a complex mapping between molecular synaptic function, and microcircuit‐level inhibitory/excitatory parameters due to intricate recurrent coupling and nonlinear effects. Predicting a priori which individual drug will specifically reproduce the parameter changes leading to escape from the ictal phase in our model is challenging. However, promising results already exist for medications impacting excitatory synaptic coupling, such as N‐methyl‐D‐aspartate receptor‐targeting ketamine^54^ and α‐amino‐3‐hydroxy‐5‐methyl‐4‐isoxazolepropionic acid receptor‐targeting perampanel,^55^ for treatment‐resistant SE. Additionally, the effects of different medications on EEG signatures of excitatory–inhibitory function could be assessed experimentally outside the context of SE, identifying key drugs that reproduce similar parameter changes.

Taken together, we provide compelling evidence that the effect of BZPs on macroscopic brain dynamics in pediatric patients with SE is primarily driven by dynamic shifts in inhibitory synaptic signaling. The observed inherent baseline variations could be leveraged through DCM to predict and optimize treatment responses in pediatric patients with SE.

### 4.1 Limitations

This analysis inadvertently relies on identifying responders and nonresponders after treatment, which may introduce a degree of bias. To minimize this, patients were classified based solely on EEG, independently of the subsequent quantitative analysis and modeling. Additionally, the small number of patients per medication type limits our ability to draw robust inferences based on individual medications.

Capturing EEG recordings during BZP administration in a clinical population presents significant challenges. Most patients receive treatment based on clinical semiology before EEG recording is initiated, often leading to the resolution of SE. Additionally, logistical challenges in timely EEG initiation during emergencies further restrict the sample size of available data. For instance, in this study, data from the 17 patients for whom BZP administration was captured on EEG were drawn from a time period during which >600 episodes of SE were treated at our hospital. Despite the small sample size, these data provide a unique opportunity to directly evaluate mechanistic hypotheses related to BZP resistance in a relevant clinical context. However, the limited sample size reduces the ability to analyze subgroup differences and robustly assess the generalizability of group‐level findings.

Several clinical factors, including the duration of SE, appropriate BZP dosing, and underlying etiology, likely influence treatment response. These factors were not specifically addressed in this study due to the limited sample size and inherent biases in the inclusion criteria. For example, detailed information on the duration of SE prior to EEG recording was not available, as most patients experienced seizure onset outside the hospital. It can be presumed, however, that the duration of SE in most patients included here likely exceeded 30 min, as inclusion required ongoing ictal activity at the time of EEG connection. At our institution, EEGs are typically initiated due to clinical concerns rather than for continuous monitoring of at‐risk patients. As a result, the onset or very early stages of SE are rarely captured on EEG.

Our study analyzes retrospective clinical EEG data specifically with the aim to test pathophysiological hypotheses from preclinical models of SE. Although this dataset allows the identification of putative group differences and computational modeling of these, the study was not powered to evaluate the predictive validity of quantitative markers of resistance. Nevertheless, our findings indicate the need for future studies to assess the predictive potential of such biomarkers and their role in improving clinical outcomes.

## AUTHOR CONTRIBUTIONS

Conceptualization: Tommaso Fedele, Richard J. Burman, Georgia Ramantani, and Richard E. Rosch. Methodology: Tommaso Fedele and Richard E. Rosch. Investigation: Tommaso Fedele, Richard J. Burman, Anne Steinberg, Giorgio Selmin, and Richard E. Rosch. Writing—original draft: Tommaso Fedele, Richard J. Burman, Georgia Ramantani, and Richard E. Rosch. Writing—review and editing: Tommaso Fedele, Richard J. Burman, Anne Steinberg, Giorgio Selmin, Georgia Ramantani, and Richard E. Rosch. Funding acquisition: Richard J. Burman, Georgia Ramantani, and Richard E. Rosch.

## CONFLICT OF INTEREST STATEMENT

The authors report no competing interests. We confirm that we have read the Journal's position on issues involved in ethical publication and affirm that this report is consistent with those guidelines.

## Supporting information


Data S1.

## Data Availability

The data that support the findings of this study are available on request from the corresponding author. The data are not publicly available due to privacy or ethical restrictions.
