# Supplementary material for: Synaptic inhibitory dynamics drive benzodiazepine response in pediatric status epilepticus
Source: Epilepsia. 2025 Apr 15;66(8):2980–94. doi: 10.1111/epi.18398 (PMC12371633; doi:10.1111/epi.18398)
Supplement: Supplementary file 1 — Data S1. [file EPI-66-2980-s001.zip › epi18398-sup-0001-TableS1-S2.docx]

| **Patient** | **Age (yrs)** | **Aetiology** | **Onset** | **Presentation** | **Type** | **Drug** | **Resp.** | **vLFP** |
| --- | --- | --- | --- | --- | --- | --- | --- | --- |
| 1 | 13 | genetic | generalized | subclinical | continuous | BZP | yes | AC |
| 2 | 9 | genetic | generalized | convulsive | continuous | BZP | yes | AC |
| 3 | 6 | genetic | focal | subclinical | continuous | BZP | yes | AC |
| 4 | 4 | genetic | focal | subclinical | continuous | BZP | yes | rMT |
| 5 | 1 | metabolic | generalized | convulsive | continuous | BZP | yes | rSMA |
| 6 | 11 | structural | focal | convulsive | continuous | BZP | yes | mC |
| 7 | 0.5 | infectious | focal | subclinical | continuous | BZP | yes | lMF |
| 8 | 1 | autoimmune | focal | subclinical | intermittent | BZP | yes | rMF |
| 9 | 4 | genetic | generalized | subclinical | continuous | BZP | no | lSF |
| 10 | 10 | genetic | focal | subclinical | continuous | BZP | no | rMF |
| 11 | 5 | genetic | focal | subclinical | continuous | BZP | no | rMO |
| 12 | 4 | genetic | generalized | subclinical | continuous | BZP | no | AC |
| 13 | 8 | genetic | generalized | subclinical | continuous | BZP | no | AC |
| 14 | 6 | genetic | generalized | subclinical | continuous | BZP | no | MC |
| 15 | 7 | metabolic | focal | subclinical | intermittent | BZP | no | MC |
| 16 | 0.6 | genetic | generalized | subclinical | intermittent | BZP | no | rIT |
| 17 | 7 | autoimmune | generalized | subclinical | continuous | BZP | no | AC |
| 18 | 9 | structural | generalized | convulsive | continuous | BAR | yes | AC |
| 19 | 13 | autoimmune | generalized | convulsive | intermittent | BAR | yes | lCal |
| 20 | 4 | autoimmune | focal | subclinical | continuous | BAR | yes | rIO |
| 21 | 2 | infectious | focal | subclinical | intermittent | BAR | yes | rCal |
| 22 | 0.3 | structural | focal | convulsive | continuous | BAR | yes | lFus |
| 23 | 3 | structural | focal | convulsive | continuous | LEV | yes | lMT |
| 24 | 8 | structural | focal | convulsive | continuous | PRO | yes | AC |
| 25 | 13 | structural | generalized | convulsive | intermittent | LEV | no | lMT |
| 26 | 10 | genetic | generalized | subclinical | continuous | VPA | no | lMF |

**Supplementary information**

**Supplementary Table 1: Cohort of paediatric patients with status epilepticus.** ‘Resp.’, Response to anti-seizure medication; ‘BAR’, barbiturate; ‘BZP’, Benzodiazepine; ‘LEV’, Levetiracetam; ‘PRO’, propofol; ‘VPA’, valproate. The location of the vLFP is described according to the AAL atlas. A, Anterior; Cal, Calcarine; C, Cingulum; F, Frontal; Fus, Fusiform; l, left; M, Middle; O, Occipital; r, right; S, Superior; SMA, Supplementary Motor Area; T, Temporal

| **Neuronal population time constant** | |
| --- | --- |
| τ_sp_ | superficial pyramidal cell time constant |
| τ_ss_ | spiny stellate time constant |
| τ_ii_ | inhibitory interneuron time constant |
| τ_dp_ | deep pyramidal cell time constant |
|  |  |
| **Coupling parameter** | |
| *γ*_sp-ss_ | superficial pyramidal cell to spiny stellate cell inhibition |
| *γ*_ii-ss_ | inhibitory interneuron to spiny stellate cells inhibition |
| *γ*_ii-dp_ | inhibitory interneuron to deep pyramidal cell inhibition |
| *γ*_ss-sp_ | spiny stellate cell to superficial pyramidal cell excitation |
| *γ*_ss-ii_ | spiny stellate cell to inhibitory interneuron excitation |
| *γ*_dp-ii_ | deep pyramidal cell to inhibitory interneuron excitation |
| *γ*_sp_ | superficial pyramidal cells self-modulation |
| *γ*_ss_ | spiny stellate cell self-modulation |
| *γ*_ii_ | inhibitory interneuron self-modulation |
| *γ*_dp_ | deep pyramidal cell self-modulation |

**Supplementary Table 2: DCM parameters.** List of synaptic parameters characterizing the CMC, which are fitted by the DCM. ‘CMC’, Canonical Micro-Circuit; ‘DCM’, Dynamical Causal Modelling.

$\dot{x_{1}}= x_{2}$

$\dot{x_{2}}=(- \gamma_{ss} S_{V_{ss}} - \gamma_{ii-ss} S_{V_{ii}}- \gamma_{sp-ss} S_{V_{sp}} - {2x}_{2}-\frac{x_{1}}{\tau_{ss}} ) \frac{1}{\tau_{ss}}$

$\dot{x_{3}}= x_{4}$

$\dot{x_{4}}=(- \gamma_{sp} S_{V_{sp}} - \gamma_{ss-sp} S_{V_{ss}}- \gamma_{sp-ss} S_{V_{sp}} - {2x}_{4}-\frac{x_{3}}{\tau_{sp}} ) \frac{1}{\tau_{sp}}$

$\dot{x_{5}}= x_{6}$

$\dot{x_{6}}=(- \gamma_{ii} S_{V_{ii}} - \gamma_{ss-ii} S_{V_{ss}}- \gamma_{dp-ii} S_{V_{dp}} - {2x}_{6}-\frac{x_{5}}{\tau_{ii}} ) \frac{1}{\tau_{ii}}$

$\dot{x_{7}}= x_{8}$

$\dot{x_{8}}=(- \gamma_{dp} S_{V_{dp}} - \gamma_{ii-dp} S_{V_{ii}} - {2x}_{8}-\frac{x_{7}}{\tau_{dp}} ) \frac{1}{\tau_{dp}}$

| **CMC state equation variables** | |
| --- | --- |
| *x_1_* | voltage (spiny stellate cells) |
| *x_2_* | conductance (spiny stellate cells) |
| *x_3_* | voltage (superficial pyramidal cells) |
| *x_4_* | conductance (superficial pyramidal cells) |
| *x_5_* | voltage (inhibitory interneuron) |
| *x_6_* | conductance (inhibitory interneuron) |
| *x_7_* | voltage (deep pyramidal cells) |
| *x_8_* | conductance (deep pyramidal cells) |
| *s_v_* | Firing rate dependent by membrane potential V |

**Supplementary Table 3: CMC state equations. ‘**CMC’, Canonical Micro-Circuit.’γ’, synaptic coupling (see Supplementary Table 2), ’τ’, synaptic time constant (see Supplementary Table 2), ‘S’, firing rate, ‘V’, membrane potential.

|  | **Hypothesis/ Hierarchical PEB model** | | |
| --- | --- | --- | --- |
|  | **Baseline difference** | **BZP response difference** | **Combination effect** |
| **t** | -19905.75 | -19901.58 | -19898.32 |
| **i** | -19476.52 | -19476.79 | **-19474.63** |
| **e** | -19508.47 | -19511.46 | -19508.08 |
| **m** | -19551.75 | -19559.56 | -19550.06 |
| **t i** | -19944.82 | -19940.18 | -19934.55 |
| **t e** | -19950.64 | -19944.78 | -19936.32 |
| **t m** | -20020.32 | -20016.07 | -20007.05 |
| **i e** | -19585.63 | -19591.31 | -19577.45 |
| **i m** | -19603.12 | -19612.06 | -19592.06 |
| **e m** | -19647.84 | -19658.49 | -19638.66 |
| **t i e** | -20025.23 | -20019.06 | -20002.64 |
| **t i m** | -20070.24 | -20068.47 | -20046.49 |
| **t e m** | -20095.44 | -20091.64 | -20070.30 |
| **i e m** | -19749.98 | -19765.42 | -19719.19 |
| **t i e m** | -20190.90 | -20194.63 | -20144.24 |

**Supplementary Table 4: Bayesian model comparison of second-level hierarchical PEB models.** Free energy values the full factorial model space coupling the three hypothesized scenarios coupled with each of the 15 subsets of synaptic parameters. ‘t’, time constants; ‘m’, self-modulatory coupling; ‘e‘, excitatory coupling; ‘i’, inhibitory coupling; ‘PEB’, parametric empirical Bayes.


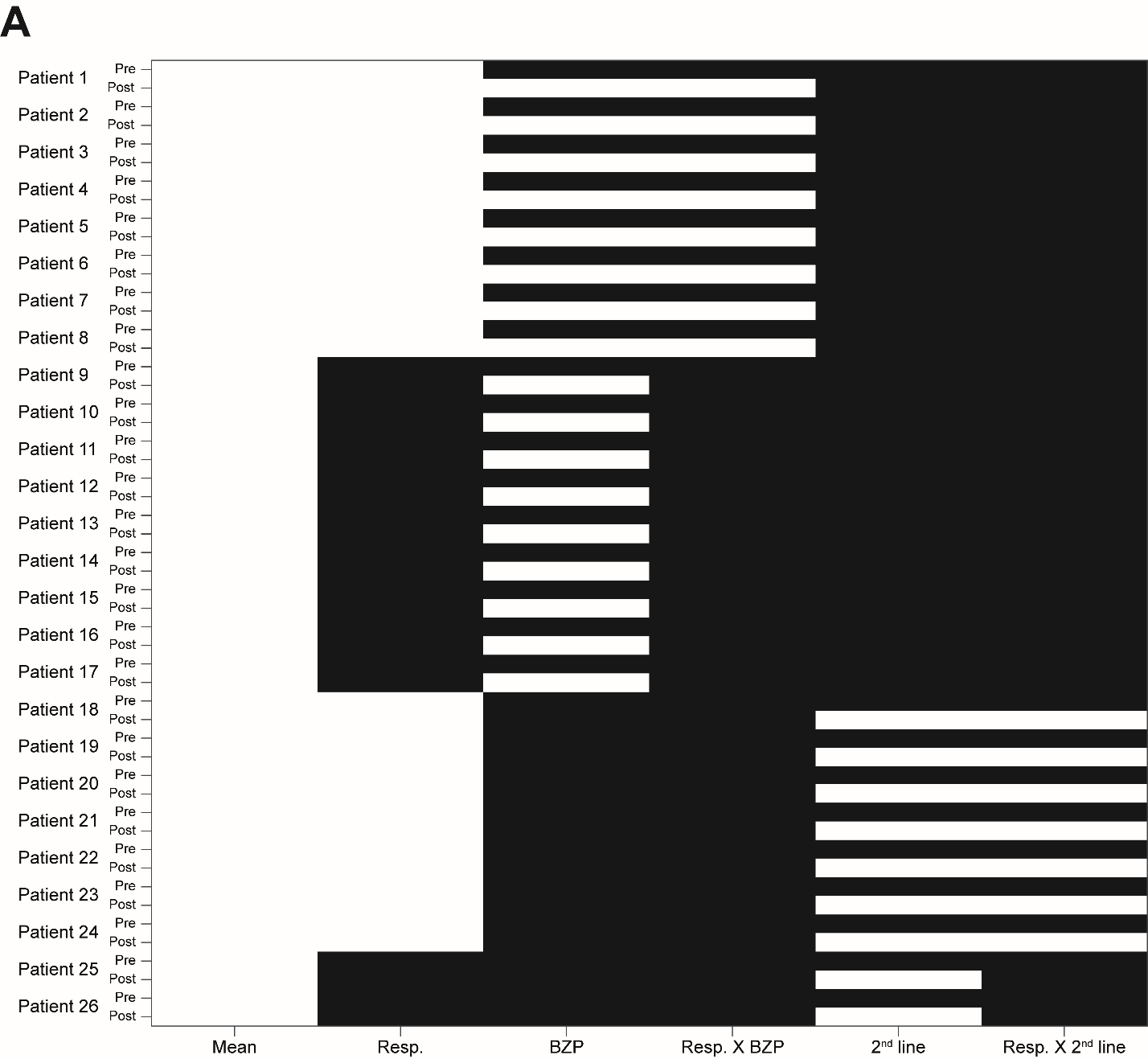


**Supplementary Figure 1: Design matrix used for hypothesis creation.** Hypothesis 1 was modelled with a design matrix comprising the main effect of responsiveness and the main effect of BZPs; hypothesis 2 was modelled with a design matrix comprising the ‘main effect of BZPs’ and the ‘interaction term’; and hypothesis 3 was modelled with a full complement of the ‘main effect of responsiveness’, the ‘main effect of BZPs’ and the ‘interaction term’. ‘BZP’, benzodiazepine; ‘Resp’, response.


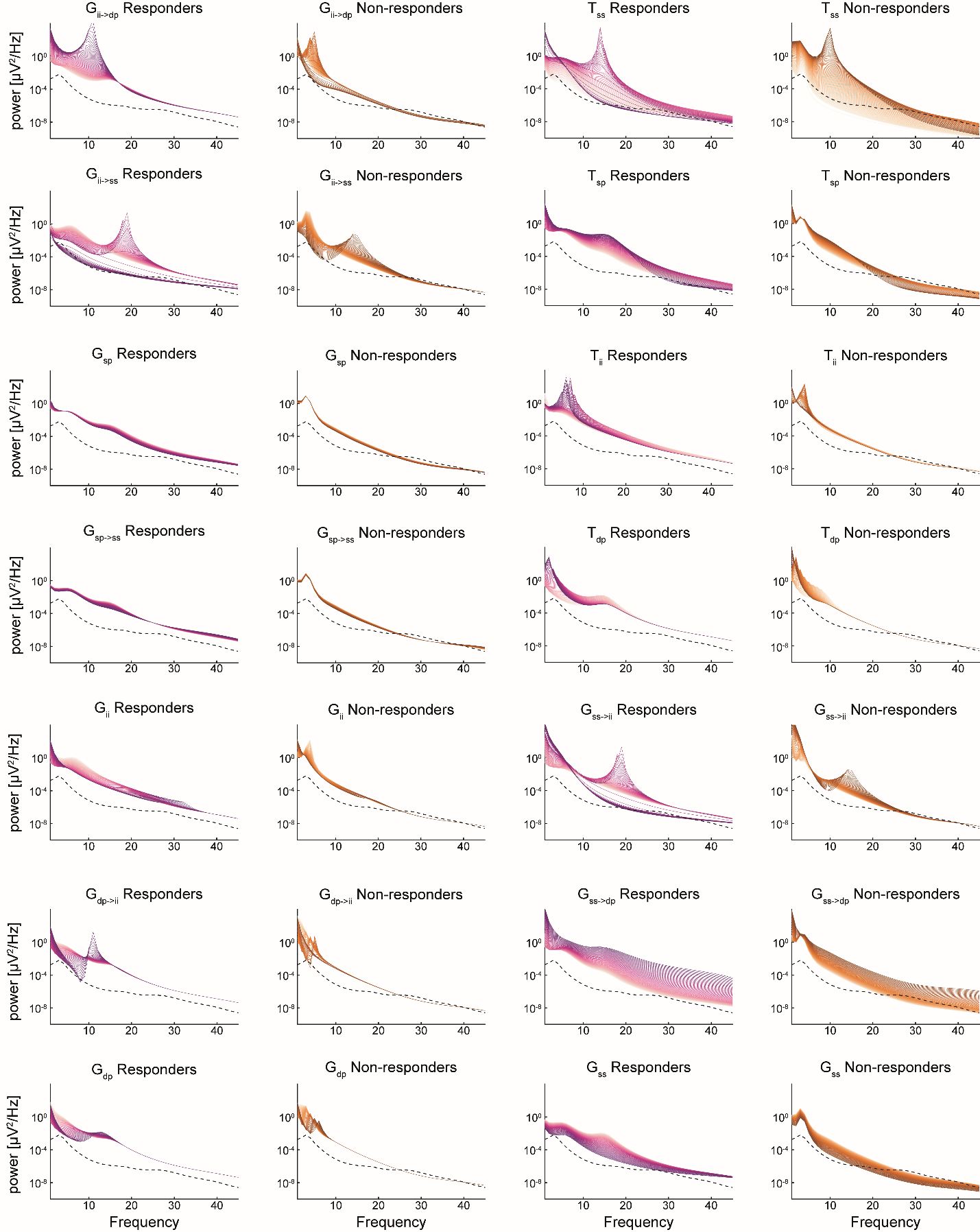


**Supplementary Figure 2: Sensitivity analysis.** Results of sensitivity analysis across conductance (G) and time constant (T) parameters for each component of the cortical microcircuit. ‘ii’, inhibitory interneuron; ‘dp’, deep pyramidal; ‘sp’, superficial pyramidal; ‘ss’, spiny stellate. In the simulation framework described in Methods, “Simulations of effects of individual synaptic parameters”, we explored the effect on the power spectra of each single DCM parameter for initial conditions set by the model for responders and non-responders. Details of the model are described in Results, “Synaptic inhibition during SE differs between BZP responders and non-responders”, and illustrated on Figure 3.


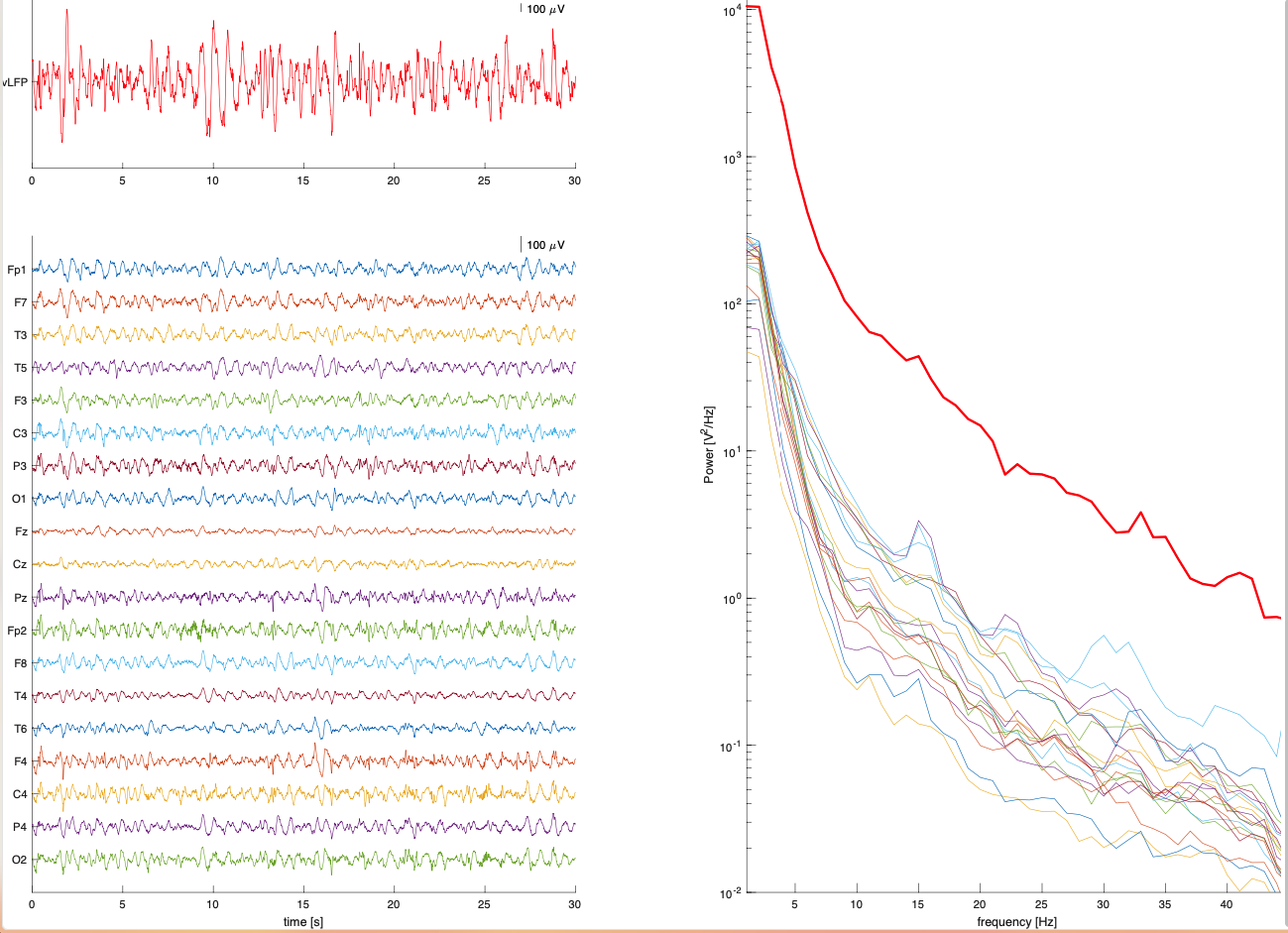


**Supplementary Figure 3: Example of dimensionality reduction in EEG. :** Left: vLFP, EEG. Right: Power spectra of the vLFP and EEG channels (Data from patient 15, focal intermittent, bottom right of Figure 1B).

| response time to benzodiazepine [seconds] | 353 ± 241 |
| --- | --- |
| response time to other medications [seconds] | 300 ± 55 |

**Supplementary Table 5: Response time in benzodiazepine responders and other medications responders.**


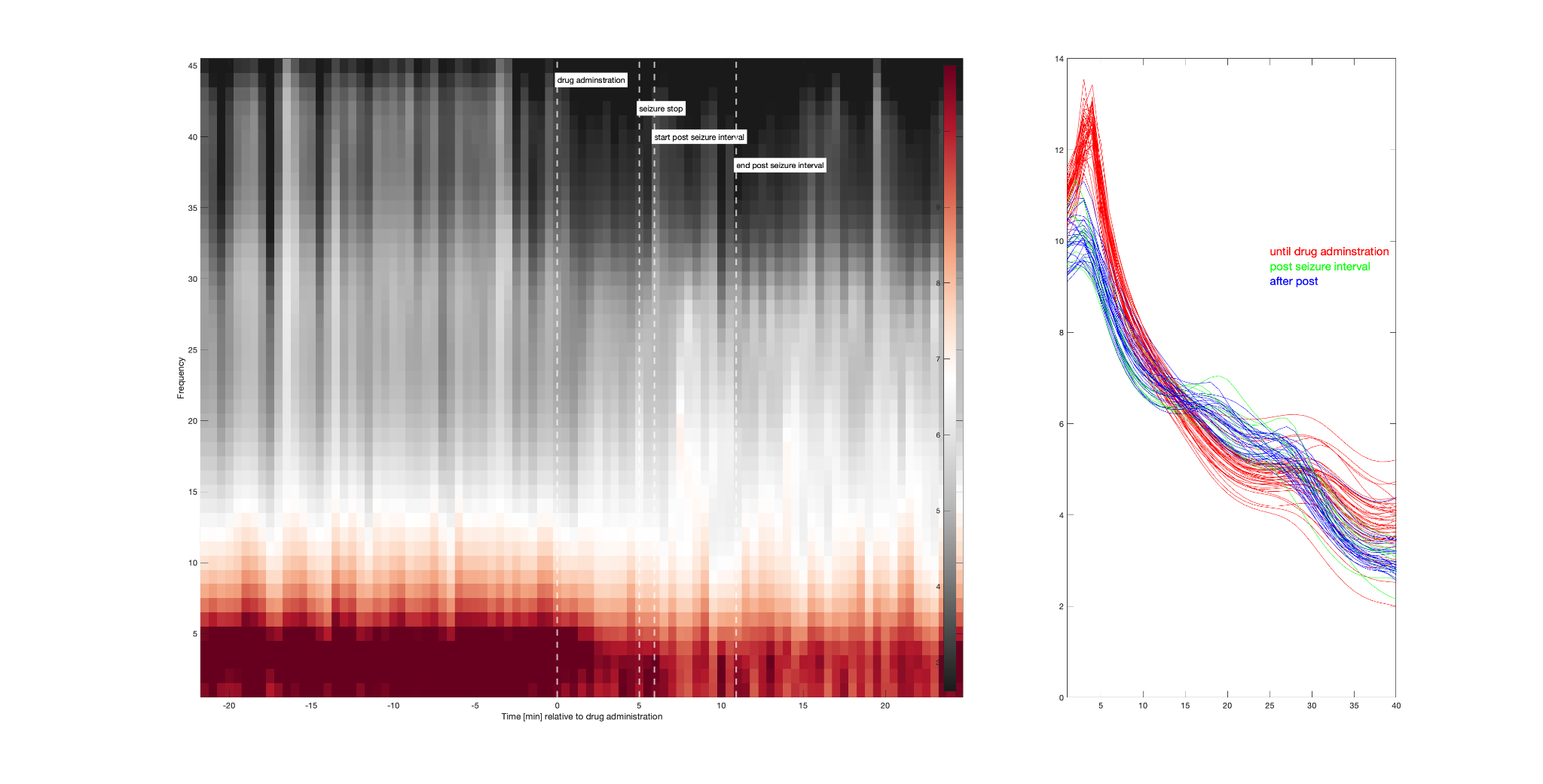


**Supplementary Figure 4. Temporal trend of spectral power in a representative responder to BZP** (Patient 3). Left panel: partial spectral density, with a resolution of 30 s. Relevant time stamps are highlighted by white dotted lines: drug administration, SE termination, start and end of the time interval considered for the 5 minute EEG post SE termination. Right panel: color-coded representation of the spectra before the drug administration (red), within the post SE termination interval (green) and following the post SE termination (blue). The power spectrum during and after SE is clearly distinguishable and it remains stable over time after SE ( green and blue spectra are similar).

| **Patient** | **Durg/responders** | **analyzed drug** | **additional drug** | **dosage** | **adminstration type** | **time stamp** | **SE termination [s]** |
| --- | --- | --- | --- | --- | --- | --- | --- |
| 18 | barbiturate | barbiturate | Temesta | 2 mg | iv | day 1 14:03:58/00:44:56 |  |
|  |  |  | **Phenobarbital** | **200 mg** |  | **day 1 01:03:46** | 261 |
| **9** | **benzodiazepine** | **benzodiazepine** | **Lorazepam** | **2,5mg** |  |  |  |
| 10 | benzodiazepine | benzodiazepine | Dormicum | 0.3 mg/kg/h | iv |  |  |
| **1** | **benzodiazepine** | **benzodiazepine** | Temesta | **3 mg** | **iv** | **day 1 06.10.2009 16:58:39/00:35:39** | **162** |
| **26** | **anticonvulsant** | **anticonvulsant** | **Keppra** | **1000 mg** | **iv** | **day 1 12.01.2012 13:54:16/00:24:41** |  |
| **2** | **benzodiazepine** | **benzodiazepine** | **Diazepam** | **10 mg** | **rectal** |  | **377** |
| **3** | **benzodiazepine** | **benzodiazepine** | **Diazepam** | **5 mg** |  | **day 1 06.01.2012 15:57:57/00:41:43** |  |
| **19** | **barbiturate** | **barbiturate** | **Phenobarbital** |  | **iv** | **day 1 13:42:00/04:41:07** | **339** |
| **11** | benzodiazepine | benzodiazepine | **Diazepam** | **10 mg** | **rectal** | **day 1 20:09:00 no mark in recording** |  |
|  |  |  | Melatonin | 10 mg | rectal | day 1 20:09:00 no mark in recording |  |
| 24 | Propofol |  | Fentanyl | 20 mg | iv | day 1 15:30:20/00:49:24 intubation |  |
|  |  |  | Dormicum | 2 mg | iv | day 1 15:30:20/00:49:24 intubation |  |
|  |  |  | **propofol** | **50 mg** | **iv** | **day 1 15:30:20/00:49:24 intubation** |  |
| 12 | benzodiazepine | benzodiazepine | Dormicum | 0.2 mg/kg/h | iv |  |  |
|  |  | **benzodiazepine** | **Midazolam** |  | **rectal** |  |  |
| 17 | benzodiazepine |  | Levetiracetam | 60 mg/kg/h | iv | day 1 11:05:00 not marked in EEG |  |
| **4** |  | **benzodiazepine** | **Diazepam** | **5 mg** | **rectal** | **day 1 11:09:00/00:07:03** | **266** |
| **5** | **benzodiazepine** | **benzodiazepine** | **Stesolid** | **5 mg** | **rectal** | **day 1 17:39:25/00:24:15** | **370** |
| 13 | benzodiazepine | benzodiazepine | Levetiracetam | 750mg | iv | day 1 09:25:34/00:02:58 |  |
|  |  |  | Levetiracetam | 750mg | iv | day 1 09:58:36/00:35:37 |  |
|  |  |  | Midazolam | 10 mg |  | day 1 08:30:00 |  |
|  |  |  | Diazepam | 10 mg rectal |  | day 1 09:00 |  |
|  |  |  | Midazolam | 5 mg | iv | day 1 10:32:00 |  |
|  |  |  | Phenytoin | 10 mg | iv | day 1 1:05:00/00:27:33 |  |
|  |  |  | Propofol 200 mg/h | 10 mg/kg/h | iv | day 1 16:05:00/00:00:00 |  |
|  |  |  | **Lorazepam(Temesta)** | **0.1 mg/kg/h** | **iv** | **day 2 11:52:38/00:35:39** |  |
| 14 | benzodiazepine | benzodiazepine | Petinutin | 2 x 150 mg |  | day 1 |  |
|  |  |  | Valproate | 2 x 420 mg |  | day 1 |  |
|  |  |  | Urbanyl | 2.5 mg |  | day 1 |  |
|  |  |  | Lacosamide (Vimpat) | 200 mg | iv | day 1. 11:03:08/00:38:41 |  |
|  |  |  | Midazolam | 2.5 gm | iv bolus | day 1 12:15:02/01:50:34 |  |
|  |  |  | **Midazolam** | **0.1 mg/kg/h** | **iv** | **day 1 12:58:02/02:33:34** |  |
| 20 | barbiturate | barbiturate | Diazepam | 10 mg | rectal | day 1 11:55:17/00:08:28 |  |
|  |  |  | Levetiracetam | 500 mg in 15' | iv | day 1 12:00:18/00:13:30 |  |
|  |  |  | Dormicum | 0.5 mg | iv | day 1 13:03:18/00:06:36 |  |
|  |  |  | Dormicum | 1.5 mg | iv | day 1 13:04:37/00:07:55 |  |
|  |  |  | Dormicum | 1 mg | iv | day 1 13:08:31/00:11:49 |  |
|  |  |  | Midazolam | 0.1 mg/kg/h | iv | day 1 13:15:40/00:18:58 |  |
|  |  |  | Levetiracetam 2 x 360 mg, (720 mg = 40 mg/kg) |  |  | day 1 |  |
|  |  |  | Levetiracetam 2x 360mg |  |  | day 2 |  |
|  |  |  | **Phenobarbital** | **250 mg in 15'** | **iv** | **day 2 20:00:00/02:11:26** |  |
| 15 | benzodiazepine | benzodiazepine | Levetiracetam 2 x 750 mg i.v. Lamotrigine 100 - 0 - 100 mg, Nitrazepam 2.5 mg in the evening Nurofen, Novalgin, Paracetamol 11.48 h, 12.07 h, 12.21 h |  |  | day 1 |  |
|  |  |  | Midazolam 2.5 mg i.v. (0.1 mg/kg/dose). | 2.5 mg | iv | day 1 11:48:56/00:25:52 |  |
|  |  |  | Midazolam 2.5 mg i.v. (0.1 mg/kg/dose). | 2.5 mg | iv | day 1 12:07:07/00:44:06 |  |
|  |  |  | Midazolam 2.5 mg i.v. (0.1 mg/kg/dose). | 2.5 mg | iv | day 1 12:21:24/00:58:20 |  |
|  |  |  | Midazolam 2.5 mg i.v. (0.1 mg/kg/dose). | 0.1 mg/kg/h | iv | day 1 12:34:00 |  |
|  |  |  | Midazolam | 2.5 mg | iv | day 1 15:00:00/03:37:50 |  |
|  |  |  | **Midazolam** | **0.2 mg/kg/h** | **iv** | **day 1 15:00:00** |  |
| 16 | benzodiazepine | benzodiazepine | Keppra | 600 mg in 15' | iv | day 1 16:29:30/00:12:48 | 803 |
|  |  |  | Midazolam | 3 mg in 6' | iv | day 1 17:01:24/00:44:43 |  |
|  |  |  | **Midazolam** | **0.1 mg/kg/h** | **iv** | **day 1 17:07:35/00:50:54** |  |
| 23 | anticonvulsant | anticonvulsant | Diazepam | 5 mg |  | day 1 09:00:00 not in the recording |  |
|  |  |  | Diazepam | 5 mg |  | day 1 09:26:00 not in the recording |  |
|  |  |  | Lorazepam (Temesta) | 1.5 mg |  | day 1 09:53:21/00:21:22 |  |
|  |  |  | **Keppra** | **500 mg in 15'** | **iv** | **day 1 10:05:12/00:33:21** |  |
| **7** | **benzodiazepine** | **benzodiazepine** | **Midazolam 0.1 mg/kg, Levetiracetam 30mg/kg,** |  |  | **day 1** | **144** |
| 16 | benzodiazepine | benzodiazepine | Anticonvulsants: Topamax 25 mg in the morning, 50 mg in the evening Phenobarbital 50 mg in the evening Keppra 2 x 200 mg Urbanyl 2.5 mg in the morning, 5 mg in the evening Medication during EEG recording: |  |  | day 1 |  |
|  |  |  | Phenytoin 130 mg at 9.30 pm | 130 mg |  | day 1 21:30:00/01:52:00 |  |
|  |  |  | **Midazolam continuous drip from 11.15 pm Start with 0.05 mg, increased to 0.2 mg/kg during the EEG recording** | **0.05 mh/kg/h - 0.2 mg/kg/h** |  | **day 1 23:15:34/03:37:17** |  |
| 17 | benzodiazepine | benzodiazepine | Midazolam | 1 mg | iv | day 1 17:59:57/00:14:51 |  |
|  |  |  | Midazolam | 1 mg | iv | day 1 18:02:13/00:17:06 |  |
|  |  |  | Levetiracetam | 700 mg in 10' | iv | day 1 18:13:33/00:28:28 |  |
|  |  |  | **Midazolam** | **1 mg** | **iv** | **day 1 18:35:55/00:50:49** |  |
| 21 | barbiturate | barbiturate | Diazepam | 5 mg | rectal | day 1 |  |
|  |  |  | Lorazepam | 0.1 mg/kg/h | iv | day 1 |  |
|  |  |  | Levetiracetam | 40 mg/kg (110mg) | iv | day 1 11:27:43/00:21:46 |  |
|  |  |  | **phenobarbital 20 mg/kg i.v.** | **20 mg/kg** | **iv** | **day 1 in shockroom** |  |
| 8 | benzodiazepine |  | Levetiracetam | 390 - 0 - 390 mg 60 mg/kg/day |  | day 1 |  |
|  |  |  | Ketamine | 20 mg |  | day 1 09:45 |  |
|  |  |  | Propofol | 50 mg |  | day 1 10:00 |  |
|  |  |  | Midazolam | 0.7 mg | iv | day 1 16:00:00/00:22:07 |  |
|  |  |  | Midazolam | 1.3 mg | iv | day 1 16:05:24/00:27:56 |  |
|  |  | benzodiazepine | **Midazolam** | **0.1 mg/kg/h** | **iv** | **day 1 16:32:26/00:55:00** |  |
| 25 | benzodiazepine |  | Levetiracetam i.v. 500 mg 1.32 p.m. | 500 mg in 20' | iv | day 1 13:31:00/00:13:33 |  |
|  |  | benzodiazepine | **Levetiracetam i.v. 2 g at 1.50 p.m. Buccolam 10 mg orally** | **1 g in 15'** | **iv** | **day 1 13:50:00/00:31:59** |  |
| 22 | barbiturate |  | Midazolam continuous drip 0.2 mg/kg/hour | 0.2 mg/kg/h | iv | day 1 |  |
|  |  |  | Levetiracetam | 100 mg | iv | day 1 11:41:26/00:42:11 |  |
|  |  |  | Fentanyl continuous drip 2 ug/kg/hour | 2 ug/kg/h | iv | day 1 |  |
|  |  |  | Phenobarbital 55 mg i.v. | 55 mg | iv | day 1 11:51:12/00:51:57 |  |
|  |  | barbiturate | **Phenobarbital 18 mg i.v. 12.50 h** | **18 mg** | **iv** | **day 1 12:50:10/01:50:58** |  |

**Supplementary Table 6: Complete clinical information on patients medications in terms of drugs, dosage and time stamps.**
